# The Relational Approach to Treating Self-Harm (RELATE): study protocol for a feasibility randomised controlled trial study of cognitive analytic therapy for adults who self-harm versus treatment at usual

**DOI:** 10.1186/s40814-024-01526-z

**Published:** 2024-07-18

**Authors:** Peter James Taylor, Isabel Adeyemi, Katie Marlow, Sarah Cottam, Zerena Airnes, Samantha Hartley, Victoria Howells, Barnaby D. Dunn, Rachel A. Elliott, Mark Hann, Cameron Latham, Catherine Robinson, Clive Turpin, Stephen Kellett

**Affiliations:** 1 Division of Psychology & Mental Health, School of Health Sciences, University of Manchester, Manchester Academic Health Sciences Centre, Manchester, UK; 2 Greater Manchester Mental Health NHS Foundation Trust, Manchester, UK; 3 Pennine Care NHS Foundation Trust, UK, Ashton-under-Lyne, UK; 4 Rotherham, Doncaster, and South Humber NHS Foundation Trust, Doncaster, UK; 5 Mood Disorders Centre, University of Exeter, Exeter, UK

**Keywords:** Self-harm, Clinical trial, Cognitive analytic therapy, RCT, Feasibility

## Abstract

**Background:**

Self-harm is a prevalent behaviour that has a major detrimental impact on a person’s life. Psychological therapies have the potential to help, but evidence of effective interventions remains limited. Access and acceptability of interventions can also be a significant challenge, with individuals either being unable to access help or having to endure long waiting lists. Cognitive analytic therapy (CAT) is a time-limited and relationally-focused psychotherapy that may provide a valuable treatment option for people who self-harm. This protocol outlines the methodology for the first feasibility randomised controlled trial (RCT) of CAT for adults that self-harm. The trial will aim to determine the feasibility, acceptability and safety of undertaking larger-scale evaluations of CAT for self-harm within an RCT context.

**Method:**

An RCT design with 1:1 allocation to CAT plus treatment as usual (TAU) or TAU alone. Participants will be adult outpatients with three or more instances of self-harm in the past year (target sample of *n* = 60). CAT will be 8 one-to-one weekly 60-min sessions plus a follow-up session up to 8 weeks after the last session. Assessments will occur at baseline, 12 weeks and 18 weeks after randomisation. Qualitative interviews with participants will gain insights into the feasibility and acceptability of CAT. Feasibility outcomes will be judged against progression criteria.

**Discussion:**

CAT may be an effective and accessible treatment option for people who self-harm, providing a more relationally orientated alternative to more behavioural therapies. The proposed feasibility RCT is an important first step in evaluating CAT as a treatment for self-harm.

**Trial registration:**

The trial was pre-registered (21/10/22) on ISR CTN (ISRCTN code: ISRCTN75661422).

**Supplementary Information:**

The online version contains supplementary material available at 10.1186/s40814-024-01526-z.

## Introduction

Self-harm, referring to intentional self-injury (e.g. cutting or hitting oneself) or self-poisoning, irrespective of suicidal intent [1, 2], is an important predictor of self-harm repetition and suicide [3–5], as well as ongoing psychological and social difficulties [6–8]. Self-harm can lead to adverse physical outcomes such as scarring and infection [9, 10] and is often an indicator of untreated psychological distress [11, 12]. It is associated with substantial treatment costs, with annual hospital costs estimated at £162 million in the UK [13]. Globally, self-harm is prevalent and a significant source of ongoing health burden [14]. In the UK, there is also evidence of increasing rates of self-harm. A nationally representative household survey found the annual rate of non-suicidal self-harm between 2000 and 2014 increased in England from 2.4 to 6.4% [15, 16].

Self-harm encompasses suicide attempts and non-suicidal self-injury. In the UK guidelines focus on self-harm as a whole [2], and some researchers have supported this position by citing the challenges in reliably ascertaining intent [17], but it has also been argued that non-suicidal behaviours differ from suicidal behaviours in important ways (intent, method, function, consequence [18]).

There remains an urgent need to develop and evaluate acceptable, effective and efficient treatments for self-harm. Whilst some systematic reviews suggest preliminary support for talking therapies [19, 20], other reviews have highlighted a lack of promising interventions [21]. Research has also highlighted poor satisfaction amongst patients with current services for self-harm [22, 23]. A comprehensive report by the UK mental health charity Samaritans highlights that many people who self-harm do not have access to effective interventions and feel they are passed around between services [23]. In the UK, primary care ‘Talking Therapy’ mental health services (formerly ‘Improving Access to Psychological Therapies’) often view people who self-harm as too complex or risky to be supported, or only able to offer therapies for other difficulties (e.g. depression or anxiety) without targeting the self-harm directly [23, 24]. Unfortunately, the same individuals may also not meet eligibility criteria for secondary care services, or when eligible, may have to endure long waiting lists for intervention. Consequently, patients report feeling they are “passed from pillar to post”, and ultimately many receive no treatment, or interventions that fail to meet their needs [23]. The need for quicker access to therapy for people who self-harm, to reduce the risk of deterioration and further self-harm, has been emphasised [25]. Brief psychological therapies that specifically target self-harm may have utility in being able to fill the gaps in existing services, offer a potentially valuable treatment choice to patients, and fit with the needs of patients.

Cognitive analytic therapy (CAT) is a time-limited (8, 16 or 24 sessions), focused, transdiagnostic, relational and integrative psychotherapy [26]. A recent meta-analysis of 28 studies showed, across conditions, improvements over time in depression, interpersonal functioning and global distress over time in participants receiving CAT (*g* = 0.74–1.05; [27]). CAT was efficacious in reducing psychological difficulties in RCTs compared with TAU (*g* = 0.53), and mixed comparators (*g* = 0.36). Another meta-analysis indexes the good acceptability of CAT, reporting a comparatively low dropout rate of 19% [28]. This rate was lower than for comparator treatments, and benchmarks well against other therapies, particularly since CAT has typically been evaluated in complex and hard-to-treat clinical populations. Specifically for self-harm, several small-scale case studies and pilots support the feasibility of CAT and CAT-informed interventions for people who self-harm [29–32].

The process and proposed mechanisms underlying CAT map well onto our understanding of self-harm. CAT views problems such as self-harm as fundamentally relational, emerging from patterns of difficult relationships with both oneself and others. Within CAT, a person’s self-concept, and the way they interact with themselves and others, develops through the internalisation of earlier social and relational experiences [26]. The concept of *reciprocal roles* (RRs) in CAT refers to dyadic internalised patterns in how someone relates to themselves and to others. An example of a positive RR might be *caring* in relation to *held/contained*. A person may internalise this RR through experiences of being cared for by others. This person may then enact this RR themselves when caring for others (adopting the *caring* pole) and may also enact this RR in relation to themselves, being able to express care for themselves during times of emotional difficulty. A negative example of an RR would be *blaming* in relation to *humiliation*. A person where this RR is especially dominant may be prone to self-blame, but also to expecting or anticipating blame from others and would feel prone to strong feelings of humiliation. CAT has a three-stage structure; firstly during reformulation the patient and therapist create narrative and diagrammatic reformulations of self-harm, during the second recognition phase, the patient uses methods to increase their reflective capacity and in the final phase active change methods are applied to facilitate change.

The CAT perspective is consistent with research indicating that a critical or hostile way of seeing and relating to oneself underlies self-harm [33–38]. A recent synthesis of existing research highlights how the interpersonal and relational context individuals exist in and enact is essential to understanding why self-harm occurs [39]. Individuals are often trapped in relational patterns where they feel disempowered and invalidated, and whilst many are ambivalent about their self-harm, they can feel they have few other ways to act or cope [39]. Self-harm itself can be overtly interpersonal in function (e.g. providing a means to communicate distress), but can also be reflective of the relationship someone has with themselves, such as where self-harm acts as a form of self-punishment, or as a form of self- defence [12, 40]. CAT aims to work with these relational dynamics and helps empower people to build more supportive relationships with others and with the self.

National guidelines currently recommend the use of cognitive behavioural therapy (CBT) for self-harm, typically of a brief duration of between four to 10 sessions [2]. A recent meta-analysis reported a treatment effect of CBT for repetition of self-harm (binary outcome) at post-treatment though not for other outcomes such as frequency of self-harm [19]. Other meta-analyses have suggested significant but small treatment effects [21]. Whilst there is evidence supporting CBT, having a range of evidence-based therapies helps maximise patient choice and satisfaction. CAT shares the time-limited, structured nature of CBT, but has a relational focus that may be particularly useful for people who self-harm, as noted above. The competencies of CAT therefore emphasise working within the therapeutic relationship. This is important given that there can be additional challenges in developing a positive working relationship in the context of self-harm [12, 41]. CAT can help both the therapist and patient to understand the way roles and patterns are being enacted in the therapeutic relationship, and thus repair the alliance ruptures that are a common feature. A recent meta-synthesis suggests that a focus on the therapeutic relationship is key in therapy for self-harm, supporting the use of relational approaches like CAT [42]. A propensity score-matched case-controlled study found that 8-session CAT within NHS ‘talking therapies’ settings was associated with comparable change to CBT for depression and anxiety, but with a lower rate of attrition [43], which is consistent with other research [28] and highlights the acceptability of the approach. A recent patient preference trial in an NHS talking therapies context also identified that patients typically preferred CAT to CBT (72% vs 28%), though this is likely influenced by many participants having already experienced CBT [44]. CAT may also offer an alternative to longer-term or more intensive interventions such as dialectical behaviour therapy (DBT).

CAT is already used in secondary care settings in the UK for experiences such as self-harm, but it is often the 24-session version that is used for patients with a BPD diagnosis [26, 45]. This format of CAT is typically used with people with complex and enduring difficulties, including those who have not benefited from other therapies, or where there are multiple interacting problems that slow useful focus being achieved. Brief 8-session CAT has been demonstrated to be suitable when therapy can achieve an early and specific focus [43]. This mirrors the recent NICE guideline recommendations for the duration of CBT for self-harm [2]. If effective, brief CAT could therefore be an accessible, more relationally-focused alternative, to more behavioural approaches like CBT and DBT.

A major challenge for expensive RCTs is recruitment and retention [46]. RELATE will indicate whether it will be feasible to undertake a large-scale trial of CAT for self-harm. In addition, RELATE will generate data on acceptability, safety, patient experience, the performance of different clinical outcomes, preliminary evidence of the promise of patient benefit and the amount of resources consumed. The central aim of the current trial is to evaluate the feasibility of an 8-session CAT for adults experiencing self-harm. The goal for a definitive trial would be to see how CAT compared to current treatment and so CAT will be compared with treatment-as-usual (TAU). Feasibility will be assessed against pre-specified progression criteria in terms of recruitment and retention of participants, and missing data on outcome measures. The trial will also determine what resource data can be collected reliably to conduct an economic evaluation. Acceptability of both the therapy and trial procedures will also be assessed via therapy adherence rates and qualitative interview data. Safety of the therapy and trial procedures will be evaluated via monitoring of Adverse Events (AEs), and participant-reported adverse experiences recorded via questionnaire and qualitative interviews. The RELATE trial has not been designed to determine treatment efficacy. Nonetheless, treatment effects on secondary clinical outcomes (e.g. self-harm behaviour and urges) will be estimated to help inform parameters required for a larger-scale trial evaluation (e.g. variance in outcome measures). In keeping with the approach of CAT, within the treatment arm of the trial, we will also monitor individual patient change on idiographic session-by-session outcomes as a further initial evaluation of the potential clinical promise of the therapy. TAU may be variable, with factors such as issues with treatment access amongst those who self-harm contributing to this [23], so this will be monitored.

## Method

### Design, randomisation and blinding

This protocol follows the reporting guidelines set out in the Standard Protocol Items: Recommendations for Interventional Trials (SPIRIT) statement ( [47]; See Supplementary Table S1). Sixty participants will be randomised at a ratio of 1:1 to either: treatment as usual (TAU) OR TAU plus the 8-session CAT intervention. A randomised block design (with random blocks of 4 or 6), stratified by the two sites, will be used, and implemented by SealedEnvelope.com. Principal Investigators (PI) will obtain allocation results for each participant, within 3 days of the completion of the baseline assessment and communicate this to the treating therapist. Non-blinded research team members will inform the participants of their treatment allocation.

**Fig. 1:**
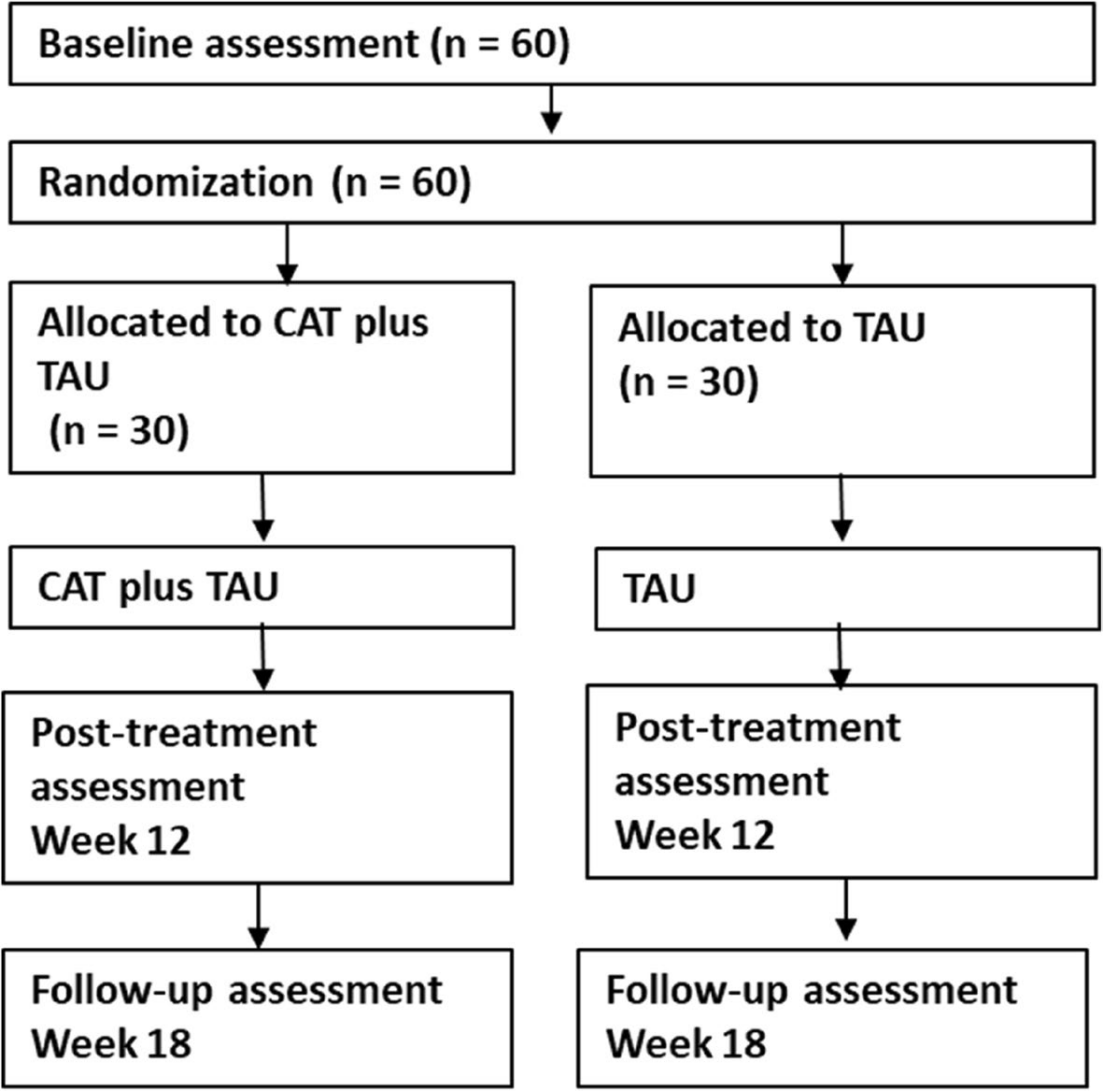
Trial flow diagram

Researchers will assess clinical outcomes at baseline and after 12 weeks post-randomisation, and 18 weeks post-randomisation. See Fig. 1 for a flow chart of trial procedures. Researchers completing assessments will be blind to allocation results. Steps will be taken to maintain blinding including reminding participants, referrers and research staff of the blinding, having therapists and researchers in separate offices and having non-blind trial documentation stored separately from other trial documentation. Blinding will be monitored, and breaks recorded. Where the blind is broken a different researcher (still blind to allocation) will complete the remaining assessments with that participant. Blinding may be intentionally broken where required in the case of medical emergencies or imminent risk. The senior management team and Trial Steering Committee (TSC) will review blind breaks to establish and implement learning and reduce further blind breaks. The trial statistician will be blind as well to minimise bias in designing or implementing the analysis.

### Participants

Participants will be adults with a recent history of self-harm recruited from community psychology and mental health services. Inclusion criteria are (i) aged 18 years or older; (ii) three or more episodes of self-harm in the past year, confirmed via the Self-Injurious Thoughts and Behaviours Interview (SITBI; [48]); (iii) can be safely seen in an outpatient clinical context in which treatment is being provided as judged by their clinical team or referrer.

Exclusion criteria are the presence of a moderate-to-severe intellectual disability (i.e. IQ < 70) as judged by their clinical team, organic cerebral disease/injury affecting receptive and expressive language comprehension as judged by their clinical team and being a current inpatient. However, if a participant becomes an inpatient during the study, they would not be withdrawn. Participants will be excluded if they are non-English speaking to the degree that the participant is unable to answer questions and give written informed consent, since no interpreter is available. Participants will also be excluded if they are already receiving another ‘high intensity’ one-to-one psychological therapy (e.g. CBT, DBT) since, if allocated to CAT, it may be problematic to engage in two different psychological therapies simultaneously. Other ‘low intensity’ forms of support, like a support group, psychoeducational or skills group, seeing a clinician for informal support or medication advice, or engaging with self-help materials, will not exclude people.

A person will not be able to participate in the researcher identifies an imminent and immediate risk to self, operationalised as the presence of active suicidal intent or planning to self-harm in the near future (e.g. next week). This is to help minimise risk to participants when they first start the trial. Where a person is excluded on these grounds, with the person’s consent, the researcher will aim to recontact them and the referrer in approximately 4 weeks time (or a time period agreed upon in collaboration with the individual) to determine if risk has subsided to a point where they are now eligible. Once a participant is in the trial, escalations in risk would not lead to them automatically being withdrawn. However, escalations in risk to self would be treated as an AE, and where trial procedures are judged to be contributing to this risk, a participant may be withdrawn from the trial (see “ Safety monitoring and reporting” section).

Experiencing a current, active episode of psychosis or mania at the time of taking consent is also an exclusion criterion, since in such instances intervention directed at the mania or psychosis may be the clinical priority. The Mini International Neuropsychiatric Interview (MINI; [49]) will be used to assess current mania and psychosis.

### Recruitment and consent

Participants will be recruited from stepped-care (NHS talking therapies) and other community-based mental health services (e.g. step 4 services) within two large NHS Foundation Trusts: Greater Manchester Mental Health NHS Foundation Trust (GMMH) and Rotherham, Doncaster, and South Humber NHS Foundation Trust (RDASH). These Trusts cover large geographical areas. GMMH covers a relatively diverse with regard to ethnicity and covers areas of high socio-economic deprivation [50, 51]. In contrast, RDASH covers a less urbanised and less diverse area but has similar levels of high socio-economic deprivation [52].

Participants will be recruited into the study in two ways. They may be referred directly by the NHS service or a person may self-refer if they are under an eligible service (e.g. community-based mental health service). Clinicians at participating recruitment sites will be able to refer potential participants who have consented to have their details shared with the research team. Service caseloads and waiting lists will also be screened for potentially eligible individuals either via a responsible clinician or administrator or by another appropriately authorised individual. Potentially eligible individuals may be identified through searches of records and would then be informed of the study via telephone or letter and asked if they wish to be referred to the study. The research team will remain in close contact with recruitment sites, attending meetings where possible to raise awareness about the study. A newsletter updating referring services about study progress will also be sent out periodically. To promote the self-referral pathway, posters advertising the study will be placed around recruitment sites.

Clinician-referred and self-referred individuals will be contacted by a researcher who will screen eligibility via self-report. Eligibility will also be further confirmed with their clinician. All potential participants who meet the initial eligibility check will be invited to a baseline assessment meeting with a researcher. This will either be in person or remotely via phone or video call. Informed consent will be taken in writing or audio-recorded. The latter will be guided by a consent script and recorded via an encrypted device.

### Cognitive analytic therapy

Therapy will be scheduled to start approximately 2 weeks after randomisation, allowing time for the logistics of scheduling appointments. We will then aim to deliver therapy within a 10-week window, finishing with the 12-week assessment. Eight 50–60-min sessions will be delivered on a weekly one-to-one basis, following a standard CAT approach [26, 43] with a focus on self-harm. The CAT will follow the established three-phase approach. Sessions 1–3 focus on developing a shared understanding of difficulties and identifying unhelpful patterns that enable and maintain self-harm, using visual (drawn) mapping of experiences and therapeutic letters to assist in this process. This is the reformulation phase of CAT. Sessions 4–5 focus on the recognition of unhelpful patterns and roles identified that maintain self-harm. This is the recognition phase of CAT. Sessions 6–8 will focus exclusively on reducing self-harm via applying ‘exits’ (i.e. change methods) and culminate in the sharing of goodbye letters between therapist and patient. All exits will be based on the individual reformulation of the patient. This is the revision phase of CAT. Following Patient and Public Involvement (PPI) discussions, a follow-up session will be negotiated with clients, up to 8 weeks after the end of therapy. This follow-up session will recap the shared understanding of the client’s difficulties, reviewing exits, recognising areas of change and troubleshooting any ongoing difficulties. Sessions can be undertaken either in person or remotely via video call. During the COVID-19 pandemic, we have seen that it is possible to deliver CAT competently in this way, and it allows participants greater flexibility and improves access [53].

The therapy will be delivered by band 7 or band 8 therapists with appropriate core professional training (e.g. clinical or counselling psychologists). The therapist will have completed post-qualification CAT training, or have completed the 1^st^ year of CAT practitioner training or equivalent. Therapists will receive fortnightly individual supervision from a CAT-accredited psychotherapist and supervisor. The competency framework for CAT will be used to help guide supervision [54]. All narrative and diagrammatic formulations will be checked for fidelity to the CAT model. Therapists will also complete a session-by-session checklist to monitor fidelity. Sessions will be audio recorded where participants consent to this, and a random 10% subset independently rated with the measure of therapist competence in CAT [55] to ascertain whether CAT is being delivered to an adequate standard. Therapists will also bring these sessions to supervision for feedback. This protocol is Template for Intervention Description and Replication (TIDieR) compliant [56].

### Treatment as usual

TAU will be the standard care offered to individuals who self-harm within the NHS trusts. This may vary and could constitute any of the following, depending on the individual, and the NHS service:

1. Structured clinical management (e.g. from a community psychiatric nurse)
2. Talking therapies (e.g. CBT, brief psychodynamic approaches) though often focused on other diagnosable problems and not self-harm specifically.
3. DBT may be offered to those with high levels of risk or psychological disturbance, or who meet criteria for personality disorder diagnoses.
4. Medication is also prescribed where clinicians judge that it may be helpful (e.g. anti-depressants).
5. Individuals may not be accessing any formal intervention at the time of the therapy (e.g. not eligible or on waiting lists) though they would still have access to crisis services and 3rd sector support.

Inconsistency in how self-harm is treated within services is problematic and is one reason why further research into what therapies work best for adults who self-harm is needed. The question of how TAU is defined in this context is a key feasibility uncertainty that will be addressed by the proposed project. To achieve this TAU received will be recorded for all participants. These data will help in determining the choice of comparator within a future definitive trial.

Referring services will be informed of treatment allocation to support their own clinical decision-making. Consequently, whilst participants in the CAT arm may also access TAU services, these are unlikely to offer participants another high-intensity structured psychological therapy if they are receiving CAT. Participants receiving CAT may be offered other interventions though, such as medication and support groups, as part of TAU, and participants will be free to seek additional intervention through other means such as private care or the 3^rd^ sector. Any such concomitant interventions will be recorded.

### Baseline characteristics

To characterise the sample, demographic information will be assessed by self-report including age, gender (including gender minorities such as being trans or non-binary), sexuality, ethnicity and marital status. Self-harm rates are elevated amongst the LGBTQ + community and hence it is important to monitor sexual orientation and gender minority status [57–59]. Health information including current and past physical and mental health diagnoses, and current and past receipt of therapy and medication will also be assessed by self-report. To further assess psychopathology, the MINI [49] will be administered at baseline and researchers will receive training in using the MINI. To minimise participant burden, only the MINI subscales relating to depression, anxiety disorders, mania, and psychosis, will be administered. Since self-harm is associated with socioeconomic deprivation [60], this will be recorded at baseline using the Indices of Multiple Deprivation (IMD; [61]). The IMD is a widely used measure that provides an indicator of socioeconomic deprivation based on participants’ postcode.

### Outcomes

#### Primary outcomes

The primary outcomes will relate to feasibility and acceptability. Table 1 presents the primary outcomes and associated progression criteria for the trial. A traffic light system (green = progress to definitive trial; amber = modification needed; red = do not progress) has been adopted to guide the decision to progress to a larger trial. A final decision concerning progression to a definitive trial will be made by the RELATE research team based on these outcomes, with support from the TSC. Acceptability will be further determined through the qualitative component of the trial. The safety of the intervention and trial procedures will be evaluated through the monitoring of AEs. This information will be supplemented with evidence of adverse experiences identified during qualitative interviews with participants. Participants receiving CAT will also be asked to complete the Adverse Effects in Psychotherapy measure [62], a self-report questionnaire that measures perceived adverse experiences that can occur in psychotherapy.

**Table 1:** Feasibility progression criteria with traffic light indicators

Outcome	Criterion	Green	Amber	Red
Recruitment	Ability to randomise 60 participants in a 12-month recruitment window	> 80%	60–79%	< 60%
Retention	Percentage of participants completing the 12-week assessment as potential primary outcome timepoint	> 80%	60–79%	< 60%
Outcome suitability	Missing data on candidate primary outcomes (SITBI; ABUSI) at 12-week assessment	< 15%	16–25%	> 25%
Adherence	Percentage of participants receiving the minimum dose of therapy (≥ 4 sessions) within 10-week treatment window	> 80%	60–79%	< 60%

*ABUSI* Alexian brothers urges to self-injure scale, *SITBI* Self-injurious thoughts and behaviours interview-short form

#### Secondary outcomes

Data pertaining to pilot clinical outcomes and putative therapy mechanisms will also be collected. Measure completeness (amount of missing data), statistical properties (e.g. variance, floor and ceiling effects), and qualitative feedback from participants will help inform the choice of primary outcomes for a future definitive trial. The anticipated primary outcome for a definitive trial is self-harm repetition, recorded via the SITBI [48], and self-harm urges, recorded via the Alexian Brothers Urges to Self-Injure scale (ABUSI; [63]), at post-treatment (12 weeks post-randomisation). Table 2 lists the outcome measures and the schedule for when they will be administered.

**Table 2:** Overview of assessment schedule

Assessment	Baseline	12 weeks	18 weeks
Demographics	x	-	-
MINI	x	-	-
SITBI	x	x	x
ABUSI	x	x	x
ESIQ	x	x	x
PSQ	x	x	x
KDS	x	x	x
IIP-32	x	x	x
EQ-5D-5L	x	x	x

*ABUSI* Alexian brothers urges to self-injure scale, *ESIQ* Experiences of self-injury questionnaire, *IIP-32* Inventory of interpersonal problems–32, *KDS* Kessler distress scale, *MINI* Mini international neuropsychiatric interview, *PSQ* Personality structure questionnaire, *SITBI* Self-injurious thoughts and behaviours interview-short form

#### Self-harm behaviour and urges

The SITBI [48] is a widely used structured interview that assesses self-harm-related thoughts and behaviour, capturing information on the occurrence, frequency and characteristics of these thoughts and behaviours over a person’s lifetime. In this trial, the modules relating to suicidal ideation, suicide attempts, and non-suicidal self-injury will be administered. Responses will be used to determine the presence and frequency of self-harm (encompassing suicide attempts and non-suicidal self-harm) over the follow-up period. We will calculate rates of suicidal behaviour and non-suicidal self-injury separately, given the argument that there can be important distinctions between these behaviours [18]. The SITBI has good inter-rater reliability and concurrent validity [48]. Urges to self-injure will be assessed using the ABUSI [63]. This is a five-item questionnaire assessing the severity of urges to self-injure over the preceding week. Scores range from 0 to 30 with higher scores indicating more severe urges to self-injure. The ABUSI shows good validity and reliability, and the single-factor structure has been supported [s63].

#### Self-harm dependence

Dependence on self-harm will be assessed with the positive beliefs subscale of the Experiences of Self-Injury Questionnaire (ESIQ; [64]). This eight-item questionnaire has scores ranging from 0 to 32, with higher scores indicating a greater perceived dependence on self-harm (i.e. greater perceived reliance and need). The factor structure, concurrent validity and reliability of this measure have been supported [64].

#### Self-concept stability

The Personality Structure Questionnaire (PSQ; [65]) will be used to assess the stability of self-concept. The questionnaire features eight items with scores ranging from 8 to 40, and higher scores indicating instability in one’s sense of self (i.e. perceived marked shifts in mood and personality). The scale is often used as a mechanistic measure for CAT [66, 67] since one hypothesised mechanism of change in CAT is the development of a more coherent and stable self-concept [26]. The reliability and validity of this measure have been supported [65, 68, 69]. Results regarding the factor structure of the questionnaire vary, but a single-factor or component solution has been most widely supported and adopted, and so will be used here [63, 66, 67].

#### Emotional distress

Self-harm is often linked to emotional distress [12]. The Kessler distress scale (K10; [70, 71]) is a widely used questionnaire of emotional distress over the past 30 days. The questionnaire has ten items, with scores ranging from 10 to 50. Higher scores indicate greater emotional distress. The scale has good reliability, concurrent validity and distinguishes well between individuals with and without mental health disorders [71, 72].

#### Interpersonal problems

Interpersonal difficulties can often be interconnected with self-harm [39]. The Inventory of Interpersonal Problems-32 (IIP-32; [73]) will be used to assess interpersonal difficulties. The questionnaire has 32 items, with scores ranging from 0 to 128. The use of a single total score as a measure of interpersonal difficulties is commonly adopted, with higher scores indicating greater difficulties. The scale has good internal reliability and was found to distinguish between the general population and psychology outpatient samples [74]. Both subscale scores and a total score have been used [74], with the latter being adopted for this trial.

#### Quality of life

The EQ-5D-5L [75] is a self-report measure of general health status. Respondents are asked to describe their health on that day in terms of mobility, self-care, engagement in activities, pain/discomfort, and depression/anxiety. A global rating of health on a scale from 0 to 100 is also requested. An index value representation of overall health can be obtained by applying a formula to respondents’ scores. A systematic review encompassing 99 papers using this tool concluded the scale had excellent psychometric properties [76].

#### Health and social care resource use

A bespoke self-report measure of health and social service use, based on the Client Service Receipt Inventory [77], designed with the involvement of experts by experience, will be completed at 18 weeks to capture this information across the course of the trial.

### Data collection

Assessment sessions will be at a place of mutual convenience for the researcher and participant. This could be the participants’ own home, a university room, a GP surgery, a mental health service, or another NHS building. We will also allow for assessment and interventions to be carried out online via video call or over the telephone in accordance with the lead Trust’s policies around remote consultation. Remote appointments allow for greater flexibility and so will help improve retention. Researchers will remain in contact with participants between assessment points, and send reminders of appointments by phone, email, and text. Participant newsletters will also be sent to help keep participants engaged in the trial. Participants will have the option to withdraw from the trial at any time or may withdraw from therapy but otherwise remain involved in the trial and still complete follow-up assessments.

During assessments, a researcher will be present to administer assessments and help participants complete questionnaires. Participants may take breaks as needed. Data will be collected on a paper Case Report Form (CRF). A copy of the CRF can be requested from the research team (excluding copyrighted material). Researchers will receive training in conducting assessments and issues in conducting assessments will be a standing item during weekly supervision meetings.

### In-therapy measures of idiographic change

As part of CAT, participants are asked to complete a personalised monitoring form within sessions that focus on the Target Problems (TPs) and Target Problem Procedures (TPPs) that are focal to self-harm, with the support of their therapist. TPs refer to a type of self-harm, whilst TPPs refer to the patterns of thinking, feeling and behaviour (called “procedures”) associated with the self-harm [26]. In the present trial a TP might be specifically self-harm, but might also be an associated underlying issue. For example, a TP might have feelings of shame, and a TPP might be a pattern of social withdrawal and self-criticism that contributes to feelings of shame that then drive self-harm. TPs and TPPs will be identified and rated in terms of recognition and revision at each session for the preceding week, using standard TP and TPP monitoring forms. These data provide an additional idiographic outcome that can be tracked across therapy sessions for those in the CAT arm.

### Qualitative process evaluation

Qualitative semi-structured interviews will be undertaken with a subset of participants from both arms of the trial. Whilst the small numbers prevent any formal stratification of the sample by factors like gender or ethnicity, purposive sampling will be adopted, with the aim of increasing the diversity with regards to trial engagement (i.e. people who withdrew from therapy or the trial), gender, ethnicity, sexual minority status and socioeconomic deprivation of those individuals invited from the larger sample to participate in the interviews. As participants become eligible for the interviews their demographic characteristics will be reviewed against the pool of participants that have already been interviewed. Where participants differ from the existing pool, reflecting some under-represented characteristics, they will be preferentially selected for the interviews. This process will be followed for participants in both trial arms.

Interviews will be guided by an interview schedule and be undertaken either in person or remotely via video call or telephone. Interviews will be audio recorded using an encrypted device. For those in the CAT arm interviews will focus on the acceptability of the therapy, perceived benefits and mechanisms of action, challenges to engagement, adverse experiences, and contextual factors seen to affect the impact of intervention. For participants in the TAU arm of the trial, interviews will concern the acceptability of trial procedures, including randomisation, assessments, and ongoing contact with the research team. The theoretical framework of acceptability will be used to help guide interviews and interpretation [78].

### Trial oversight

The Trial Management Group (TMG) will oversee the running of the trial, consisting of PIs and co-investigators. This group will meet monthly. Researchers will be invited to weekly supervision meetings with the trial PIs, supporting the week-to-week running of the trial. The TSC will provide independent advice, guidance and oversight. The TSC has six independent members and two non-independent members including clinicians, experts in self-harm research, experts in trial design and evaluation, and individuals with relevant lived experience, and will meet at least bi-annually.

### Patient and public involvement

The trial was designed through consultation with people with lived experience of self-harm. The co-investigator team includes an individual with lived experience of self-harm and mental health services, who will be able to draw on these experiences in guiding the trial and is a co-author on this paper. An advisory group of four individuals with lived experience of self-harm has been set up and will meet four times a year, providing further advice and guidance about the running of the trial.

### Safety monitoring and reporting

AEs refer to any untoward medical or psychological occurrence and will be routinely monitored and recorded during the course of the trial. Self-harm is an anticipated AE given the nature of the trial and will be asked about during assessment meetings. Given the small scale of this trial, there will not be a separate Data Monitoring and Ethics Committee (DMEC). Serious Adverse Events (SAEs) will be reported to the TSC, who will be asked to decide whether the event may have been caused by trial procedures (i.e. an adverse reaction). The TSC may convene extraordinary meetings to discuss SAEs where required. Where a possible serious adverse reaction is identified, the next steps will be agreed upon in discussion with the TSC, trial sponsor, and ethics committee, and may include removing the participant from the trial, pausing the trial, or stopping the trial.

### Sample size

We will aim to recruit 60 people for the study (30 per trial arm). This number will be sufficient to estimate key parameters to inform a future definitive trial, (e.g. the standard deviation of key outcomes; the attrition rate), to an adequate degree of precision [79]. For the qualitative process evaluation, a target of 15 participants from the CAT arm of the trial, and 5 participants from the TAU arm will be sought. Drawing on the principles of information power [80] we note the sample specificity is dense and the qualitative process evaluation has a relatively narrow aim, but there is no guiding theory and a cross-case analysis is sought. A sample size of 20 overall therefore appears adequate. The imbalance between CAT and TAU arms reflects our particular interest in the experience of the therapy itself, and the anticipation that experiences of the therapy may be more complex and varied, than experiences of being in the TAU arm, requiring a larger sub-sample. The idiographic change analysis of TP and TPs will be based on the 30 participants in the CAT arm.

### Data management

Data management will be guided by a data management and monitoring plan. A spreadsheet will be used to track participant progress through the trial. Paper CRF will be scanned to provide electronic copies, and data entered into an electronic dataset as soon as possible. The dataset will be set up to minimise data entry errors so that inadmissible or out-of-range values cannot be entered. A random 10% subset of data will be independently re-entered and checked against the original to identify errors. Where the rate of error exceeds an acceptable threshold (1%) action will be taken. This will vary depending on the perceived cause of the errors but may include re-entering portions of the data and further checks. Audio recordings of qualitative interviews will be transcribed, with personally identifiable information (e.g. names and locations) removed from transcripts.

Personally identifiable data will be stored separately from other study data. An ID code will be used to identify and link data. All electronic data will be stored securely on password-protected NHS computer drives accessible only by the study team. Hard data will be stored securely within locked NHS premises. All study research staff will receive training in data management. Upon completion of the trial personally identifiable data will be destroyed (with the exception of consent forms and recordings that will be retained for 5 years) leaving only anonymised data.

### Data analysis

#### Quantitative data analysis

Data analysis will follow an Intention-To-Treat (ITT) protocol. We will present descriptive data relating to participant recruitment, retention and attrition in a CONSORT flow chart [81]. The treatment refusal and treatment attrition rates will be calculated (with confidence intervals) and reasons for drop-out will be recorded where possible. We will also assess the rates of missing data on specific questionnaires. We will summarise, as appropriate (e.g. central tendency, variance, range), data for all potential outcome measures, overall and by group. As the study is not formally powered, hypothesis testing to determine intervention effectiveness will not be conducted: however, we will report confidence intervals for the between-trial arm coefficient from appropriate regression analyses to investigate the ‘promise’ of CAT. The TP and TPP data, collected for those in the CAT arm, will be evaluated using single-case design methods [82], including visual graphing of change over time, and non-overlap statistics to examine how outcomes change between the three phases of the therapy. Mean imputation of individual scale items where overall missing items on the scale are < 20% will be adopted in order to generate scale totals. No other imputation will be used. A more detailed statistical analysis plan will the developed with the trial statistician and reviewed by the TSC.

#### Qualitative analysis

Qualitative data will be transcribed verbatim and analysed via reflexive thematic analysis [83, 84]. Analyses will use an inductive approach involving the initial coding of key concepts and ideas within the transcripts, which are then used to identify broader themes that are apparent across the dataset. A critical realist perspective will be adopted [85]. This allows for the recognition of a shared reality underlying participants’ responses, whilst also holding in mind the potential impact of the research teams’ assumptions and the context of the work.

#### Health economics

A full economic evaluation is not planned but information relevant to the economic evaluation of the therapy will be summarised. Following the NICE Reference Case, EQ-5D-5L responses will be converted to EQ-5D-3L utility values using the method by Hernández Alava and colleagues [86]. Quality-adjusted life years (QALYs) per participant will be calculated by the area under the curve method. Health and social care resources used by participants will be captured, as described above, and summarised. These data will be used to inform the full economic evaluation design within a subsequent definitive trial.

### Ethics and auditing

The trial has received ethical approval from an NHS research ethics committee (Greater Manchester West REC; ID: 318068). Proposed modifications to the trial that require an ethical amendment will first be reviewed by the TSC, and if required, the study sponsor and funder. No audits are planned but the study may be audited at any time by relevant agencies including the trial sponsor and institutional partners. The TSC and sponsor may request audits. Protocol adherence will be monitored by the TMG and deviations from protocol recorded.

### Dissemination

The trial results will be disseminated via peer-reviewed publications and conference presentations. Lay summaries and an infographic will also be developed and shared via social media and on the websites of institutional project partners, with the aim of engaging the wider public. The patient and public involvement advisory group will be involved in the development of these materials. Press releases will be issued in order to generate media interest. A stakeholder dissemination and discussion event will also be organised. This event will be open to key project stakeholders (clinicians, service managers, referrers, people affected by self-harms and carers) and will involve sharing the research findings and generating discussion about the implications of these results. The TMG will agree on the authorship of publications in advance of submission, following international guidelines (e.g. https://www.apa.org/research/responsible/publication). Disputes concerning authorship will be resolved through discussion with the TSC.

## Discussion

Self-harm is a prevalent problem in the community that represents globally a major ongoing burden for people. CAT had shown initial promise as a time-limited therapy for adults who struggle with self-harm, and so may provide an effective and efficient alternative to more intensive approaches like DBT. However, the evidence for CAT as a treatment for self-harm is lacking, and controlled clinical trials are therefore needed [87]. The RELATE trial will determine the feasibility of larger-scale evaluations of CAT for self-harm. The qualitative process evaluation will also provide valuable insight into the experience of receiving CAT for self-harm and help elucidate mechanisms of action. The idiographic analysis of change for CAT participants of TP and TPPs related to self-harm will illuminate the pattern of the change process over the three phases of the approach. If trial progression criteria are met, the next step would be to conduct a definitive RCT focused on the clinical efficacy and cost-effectiveness of CAT.

### Supplementary Information

Supplementary Material 1. Supplementary Table S1. SPIRT Checklist.

## Data Availability

An anonymised version of the final trial dataset will be made available to other researchers following the publication of the study.
